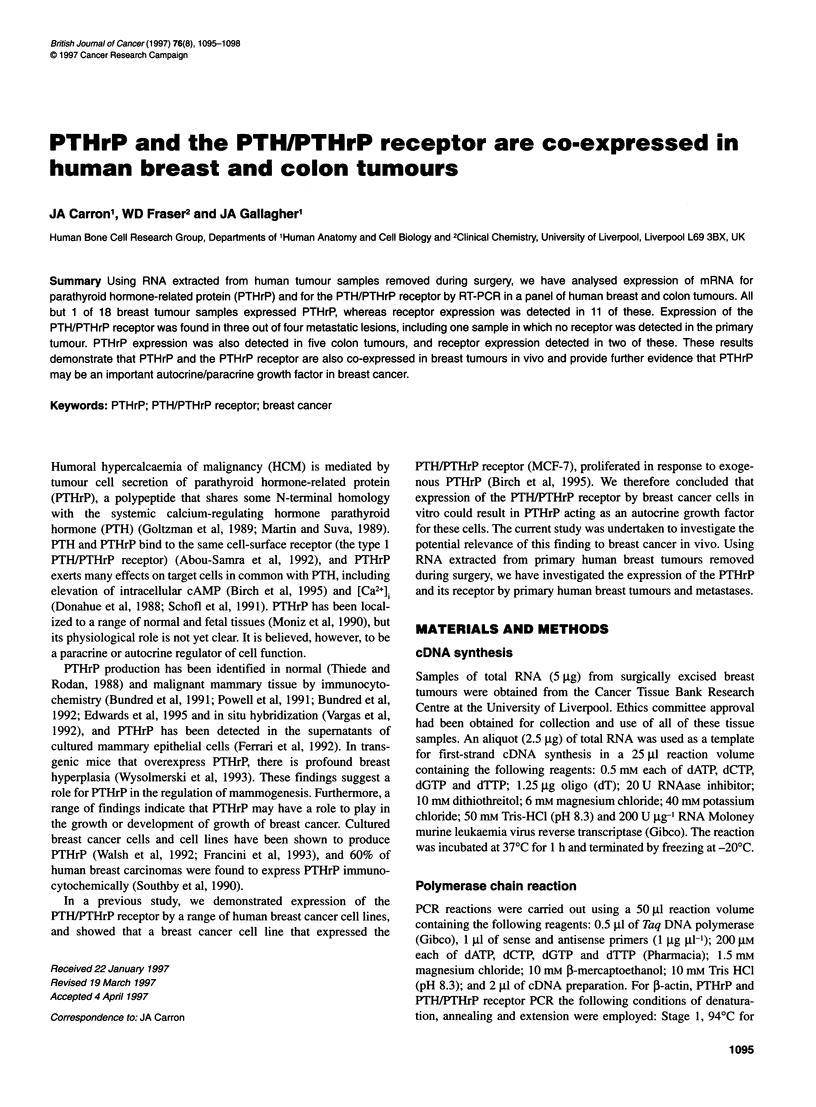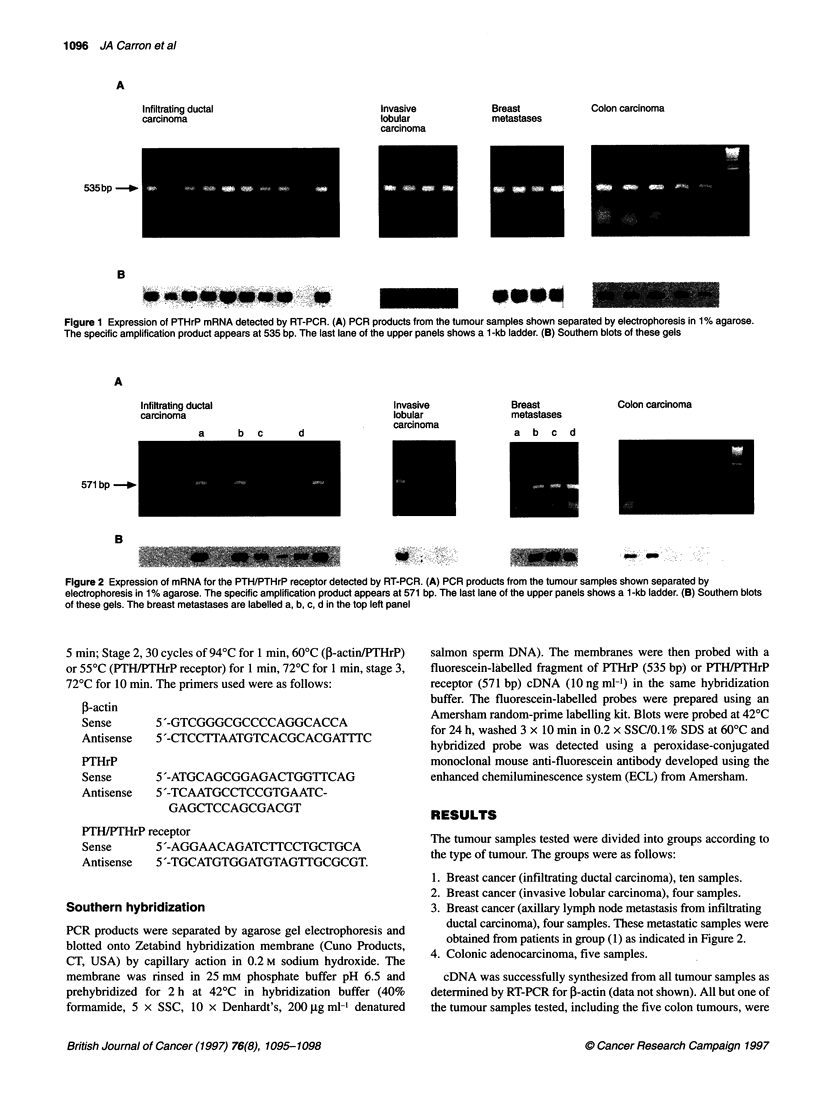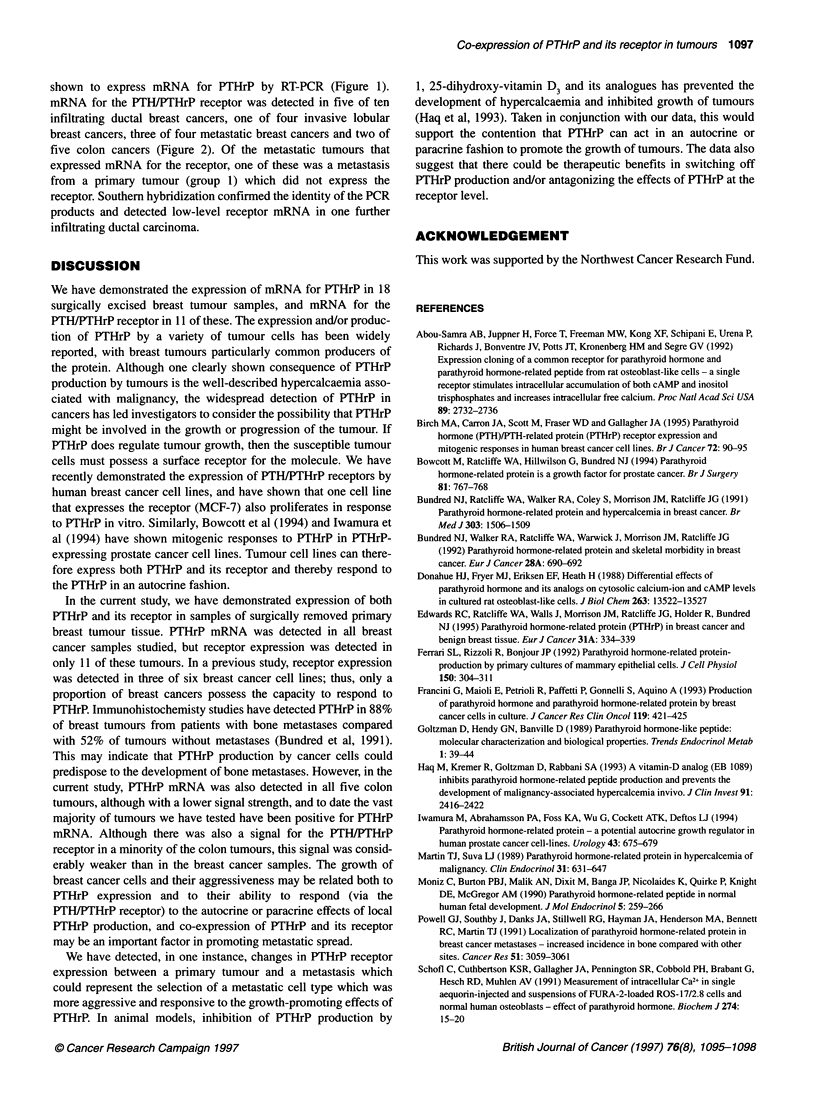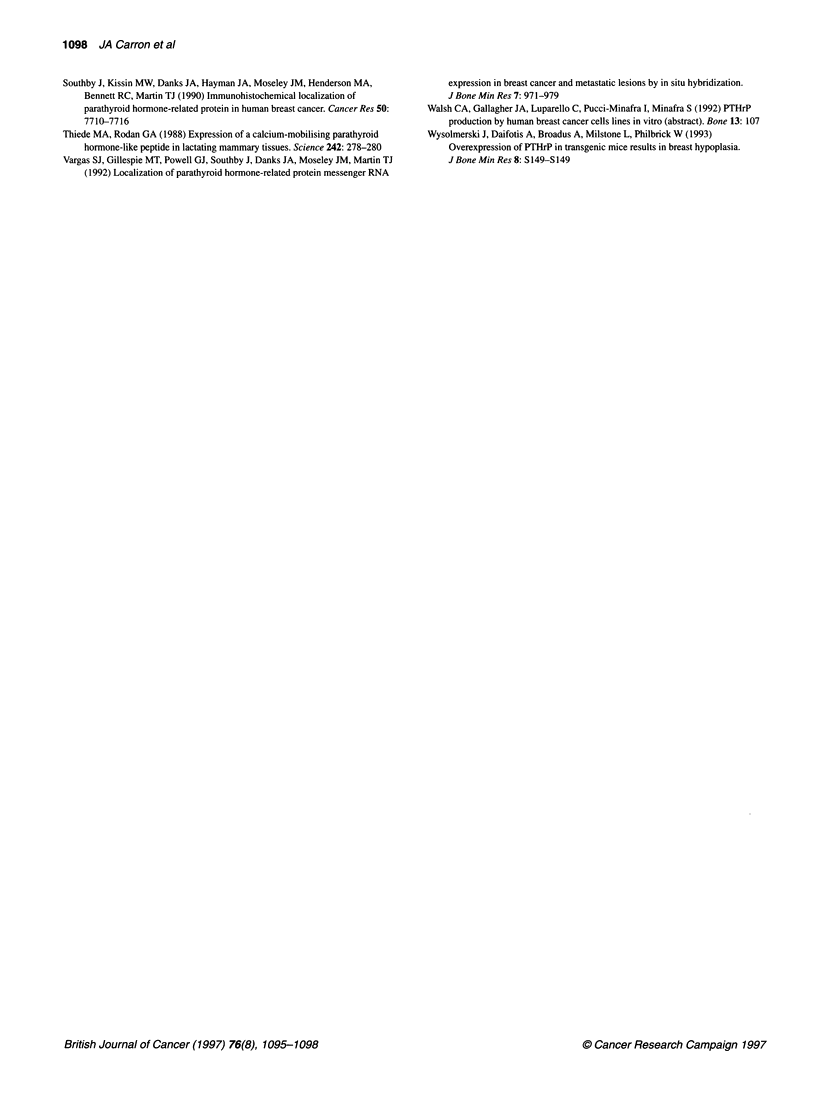# PTHrP and the PTH/PTHrP receptor are co-expressed in human breast and colon tumours.

**DOI:** 10.1038/bjc.1997.513

**Published:** 1997

**Authors:** J. A. Carron, W. D. Fraser, J. A. Gallagher

**Affiliations:** Human Bone Cell Research Group, Department of Human Anatomy, University of Liverpool, UK.

## Abstract

**Images:**


British Journal of Cancer (1997) 76(8), 1095–1098
© 1997 Cancer Research Campaign

### PTHrP and the PTH/PTHrP receptor are co-expressed in human breast and colon tumours

**JA Carron[1], WD Fraser[2] and JA Gallagher[1]**

Human Bone Cell Research Group, Departments of [1]Human Anatomy and Cell Biology and [2]Clinical Chemistry, University of Liverpool, Liverpool L69 3BX, UK

**Summary** Using RNA extracted from human tumour samples removed during surgery, we have analysed expression of mRNA for parathyroid hormone-related protein (PTHrP) and for the PTH/PTHrP receptor by RT-PCR in a panel of human breast and colon tumours. All but 1 of 18 breast tumour samples expressed PTHrP, whereas receptor expression was detected in 11 of these. Expression of the PTH/PTHrP receptor was found in three out of four metastatic lesions, including one sample in which no receptor was detected in the primary tumour. PTHrP expression was also detected in five colon tumours, and receptor expression detected in two of these. These results demonstrate that PTHrP and the PTHrP receptor are also co-expressed in breast tumours in vivo and provide further evidence that PTHrP may be an important autocrine/paracrine growth factor in breast cancer.

**Keywords:** PTHrP; PTH/PTHrP receptor; breast cancer

Humoral hypercalcaemia of malignancy (HCM) is mediated by tumour cell secretion of parathyroid hormone-related protein (PTHrP), a polypeptide that shares some N-terminal homology with the systemic calcium-regulating hormone parathyroid hormone (PTH) (Goltzman et al, 1989; Martin and Suva, 1989). PTH and PTHrP bind to the same cell-surface receptor (the type 1 PTH/PTHrP receptor) (Abou-Samra et al, 1992), and PTHrP exerts many effects on target cells in common with PTH, including elevation of intracellular cAMP (Birch et al, 1995) and $[Ca^{2+}]_i$ (Donahue et al, 1988; Schofl et al, 1991). PTHrP has been localized to a range of normal and fetal tissues (Moniz et al, 1990), but its physiological role is not yet clear. It is believed, however, to be a paracrine or autocrine regulator of cell function.

PTHrP production has been identified in normal (Thiede and Rodan, 1988) and malignant mammary tissue by immunocytochemistry (Bundred et al, 1991; Powell et al, 1991; Bundred et al, 1992; Edwards et al, 1995 and in situ hybridization (Vargas et al, 1992), and PTHrP has been detected in the supernatants of cultured mammary epithelial cells (Ferrari et al, 1992). In transgenic mice that overexpress PTHrP, there is profound breast hyperplasia (Wysolmerski et al, 1993). These findings suggest a role for PTHrP in the regulation of mammogenesis. Furthermore, a range of findings indicate that PTHrP may have a role to play in the growth or development of growth of breast cancer. Cultured breast cancer cells and cell lines have been shown to produce PTHrP (Walsh et al, 1992; Francini et al, 1993), and 60% of human breast carcinomas were found to express PTHrP immunocytochemically (Southby et al, 1990).

In a previous study, we demonstrated expression of the PTH/PTHrP receptor by a range of human breast cancer cell lines, and showed that a breast cancer cell line that expressed the PTH/PTHrP receptor (MCF-7), proliferated in response to exogenous PTHrP (Birch et al, 1995). We therefore concluded that expression of the PTH/PTHrP receptor by breast cancer cells in vitro could result in PTHrP acting as an autocrine growth factor for these cells. The current study was undertaken to investigate the potential relevance of this finding to breast cancer in vivo. Using RNA extracted from primary human breast tumours removed during surgery, we have investigated the expression of the PTHrP and its receptor by primary human breast tumours and metastases.

*Received 22 January 1997*
*Revised 19 March 1997*
*Accepted 4 April 1997*

*Correspondence to:* JA Carron

### MATERIALS AND METHODS

### cDNA synthesis

Samples of total RNA (5 µg) from surgically excised breast tumours were obtained from the Cancer Tissue Bank Research Centre at the University of Liverpool. Ethics committee approval had been obtained for collection and use of all of these tissue samples. An aliquot (2.5 µg) of total RNA was used as a template for first-strand cDNA synthesis in a 25 µl reaction volume containing the following reagents: 0.5 mM each of dATP, dCTP, dGTP and dTTP; 1.25 µg oligo (dT); 20 U RNAase inhibitor; 10 mM dithiothreitol; 6 mM magnesium chloride; 40 mM potassium chloride; 50 mM Tris-HCl (pH 8.3) and 200 U $µg^{-1}$ RNA Moloney murine leukaemia virus reverse transcriptase (Gibco). The reaction was incubated at 37°C for 1 h and terminated by freezing at –20°C.

### Polymerase chain reaction

PCR reactions were carried out using a 50 µl reaction volume containing the following reagents: 0.5 µl of *Taq* DNA polymerase (Gibco), 1 µl of sense and antisense primers (1 µg $µl^{-1}$); 200 µM each of dATP, dCTP, dGTP and dTTP (Pharmacia); 1.5 mM magnesium chloride; 10 mM β-mercaptoethanol; 10 mM Tris HCl (pH 8.3); and 2 µl of cDNA preparation. For β-actin, PTHrP and PTH/PTHrP receptor PCR the following conditions of denaturation, annealing and extension were employed: Stage 1, 94°C for

**Figure 1** Expression of PTHrP mRNA detected by RT-PCR. (**A**) PCR products from the tumour samples shown separated by electrophoresis in 1% agarose. The specific amplification product appears at 535 bp. The last lane of the upper panels shows a 1-kb ladder. (**B**) Southern blots of these gels

**Figure 2** Expression of mRNA for the PTH/PTHrP receptor detected by RT-PCR. (**A**) PCR products from the tumour samples shown separated by electrophoresis in 1% agarose. The specific amplification product appears at 571 bp. The last lane of the upper panels shows a 1-kb ladder. (**B**) Southern blots of these gels. The breast metastases are labelled a, b, c, d in the top left panel

5 min; Stage 2, 30 cycles of 94°C for 1 min, 60°C (β-actin/PTHrP) or 55°C (PTH/PTHrP receptor) for 1 min, 72°C for 1 min, stage 3, 72°C for 10 min. The primers used were as follows:

| | |
|---|---|
| β-actin | |
| Sense | 5′-GTCGGGCGCCCCAGGCACCA |
| Antisense | 5′-CTCCTTAATGTCACGCACGATTTC |
| PTHrP | |
| Sense | 5′-ATGCAGCGGAGACTGGTTCAG |
| Antisense | 5′-TCAATGCCTCCGTGAATC-GAGCTCCAGCGACGT |
| PTH/PTHrP receptor | |
| Sense | 5′-AGGAACAGATCTTCCTGCTGCA |
| Antisense | 5′-TGCATGTGGATGTAGTTGCGCGT. |

### Southern hybridization

PCR products were separated by agarose gel electrophoresis and blotted onto Zetabind hybridization membrane (Cuno Products, CT, USA) by capillary action in 0.2 M sodium hydroxide. The membrane was rinsed in 25 mM phosphate buffer pH 6.5 and prehybridized for 2 h at 42°C in hybridization buffer (40% formamide, 5 × SSC, 10 × Denhardt's, 200 μg $ml^{-1}$ denatured salmon sperm DNA). The membranes were then probed with a fluorescein-labelled fragment of PTHrP (535 bp) or PTH/PTHrP receptor (571 bp) cDNA (10 ng $ml^{-1}$) in the same hybridization buffer. The fluorescein-labelled probes were prepared using an Amersham random-prime labelling kit. Blots were probed at 42°C for 24 h, washed 3 × 10 min in 0.2 × SSC/0.1% SDS at 60°C and hybridized probe was detected using a peroxidase-conjugated monoclonal mouse anti-fluorescein antibody developed using the enhanced chemiluminescence system (ECL) from Amersham.

### RESULTS

The tumour samples tested were divided into groups according to the type of tumour. The groups were as follows:

1. Breast cancer (infiltrating ductal carcinoma), ten samples.
2. Breast cancer (invasive lobular carcinoma), four samples.
3. Breast cancer (axillary lymph node metastasis from infiltrating ductal carcinoma), four samples. These metastatic samples were obtained from patients in group (1) as indicated in Figure 2.
4. Colonic adenocarcinoma, five samples.

cDNA was successfully synthesized from all tumour samples as determined by RT-PCR for β-actin (data not shown). All but one of the tumour samples tested, including the five colon tumours, were

 © Cancer Research Campaign 1997

shown to express mRNA for PTHrP by RT-PCR (Figure 1). mRNA for the PTH/PTHrP receptor was detected in five of ten infiltrating ductal breast cancers, one of four invasive lobular breast cancers, three of four metastatic breast cancers and two of five colon cancers (Figure 2). Of the metastatic tumours that expressed mRNA for the receptor, one of these was a metastasis from a primary tumour (group 1) which did not express the receptor. Southern hybridization confirmed the identity of the PCR products and detected low-level receptor mRNA in one further infiltrating ductal carcinoma.

### DISCUSSION

We have demonstrated the expression of mRNA for PTHrP in 18 surgically excised breast tumour samples, and mRNA for the PTH/PTHrP receptor in 11 of these. The expression and/or production of PTHrP by a variety of tumour cells has been widely reported, with breast tumours particularly common producers of the protein. Although one clearly shown consequence of PTHrP production by tumours is the well-described hypercalcaemia associated with malignancy, the widespread detection of PTHrP in cancers has led investigators to consider the possibility that PTHrP might be involved in the growth or progression of the tumour. If PTHrP does regulate tumour growth, then the susceptible tumour cells must possess a surface receptor for the molecule. We have recently demonstrated the expression of PTH/PTHrP receptors by human breast cancer cell lines, and have shown that one cell line that expresses the receptor (MCF-7) also proliferates in response to PTHrP in vitro. Similarly, Bowcott et al (1994) and Iwamura et al (1994) have shown mitogenic responses to PTHrP in PTHrP-expressing prostate cancer cell lines. Tumour cell lines can therefore express both PTHrP and its receptor and thereby respond to the PTHrP in an autocrine fashion.

In the current study, we have demonstrated expression of both PTHrP and its receptor in samples of surgically removed primary breast tumour tissue. PTHrP mRNA was detected in all breast cancer samples studied, but receptor expression was detected in only 11 of these tumours. In a previous study, receptor expression was detected in three of six breast cancer cell lines; thus, only a proportion of breast cancers possess the capacity to respond to PTHrP. Immunohistochemisty studies have detected PTHrP in 88% of breast tumours from patients with bone metastases compared with 52% of tumours without metastases (Bundred et al, 1991). This may indicate that PTHrP production by cancer cells could predispose to the development of bone metastases. However, in the current study, PTHrP mRNA was also detected in all five colon tumours, although with a lower signal strength, and to date the vast majority of tumours we have tested have been positive for PTHrP mRNA. Although there was also a signal for the PTH/PTHrP receptor in a minority of the colon tumours, this signal was considerably weaker than in the breast cancer samples. The growth of breast cancer cells and their aggressiveness may be related both to PTHrP expression and to their ability to respond (via the PTH/PTHrP receptor) to the autocrine or paracrine effects of local PTHrP production, and co-expression of PTHrP and its receptor may be an important factor in promoting metastatic spread.

We have detected, in one instance, changes in PTHrP receptor expression between a primary tumour and a metastasis which could represent the selection of a metastatic cell type which was more aggressive and responsive to the growth-promoting effects of PTHrP. In animal models, inhibition of PTHrP production by 1, 25-dihydroxy-vitamin $D_3$ and its analogues has prevented the development of hypercalcaemia and inhibited growth of tumours (Haq et al, 1993). Taken in conjunction with our data, this would support the contention that PTHrP can act in an autocrine or paracrine fashion to promote the growth of tumours. The data also suggest that there could be therapeutic benefits in switching off PTHrP production and/or antagonizing the effects of PTHrP at the receptor level.

### ACKNOWLEDGEMENT

This work was supported by the Northwest Cancer Research Fund.

### REFERENCES

Abou-Samra AB, Juppner H, Force T, Freeman MW, Kong XF, Schipani E, Urena P, Richards J, Bonventre JV, Potts JT, Kronenberg HM and Segre GV (1992) Expression cloning of a common receptor for parathyroid hormone and parathyroid hormone-related peptide from rat osteoblast-like cells – a single receptor stimulates intracellular accumulation of both cAMP and inositol trisphosphates and increases intracellular free calcium. *Proc Natl Acad Sci USA* **89**: 2732–2736

Birch MA, Carron JA, Scott M, Fraser WD and Gallagher JA (1995) Parathyroid hormone (PTH)/PTH-related protein (PTHrP) receptor expression and mitogenic responses in human breast cancer cell lines. *Br J Cancer* **72**: 90–95

Bowcott M, Ratcliffe WA, Hillwilson G, Bundred NJ (1994) Parathyroid hormone-related protein is a growth factor for prostate cancer. *Br J Surgery* **81**: 767–768

Bundred NJ, Ratcliffe WA, Walker RA, Coley S, Morrison JM, Ratcliffe JG (1991) Parathyroid hormone-related protein and hypercalcemia in breast cancer. *Br Med J* **303**: 1506–1509

Bundred NJ, Walker RA, Ratcliffe WA, Warwick J, Morrison JM, Ratcliffe JG (1992) Parathyroid hormone-related protein and skeletal morbidity in breast cancer. *Eur J Cancer* **28A**: 690–692

Donahue HJ, Fryer MJ, Eriksen EF, Heath H (1988) Differential effects of parathyroid hormone and its analogs on cytosolic calcium-ion and cAMP levels in cultured rat osteoblast-like cells. *J Biol Chem* **263**: 13522–13527

Edwards RC, Ratcliffe WA, Walls J, Morrison JM, Ratcliffe JG, Holder R, Bundred NJ (1995) Parathyroid hormone-related protein (PTHrP) in breast cancer and benign breast tissue. *Eur J Cancer* **31A**: 334–339

Ferrari SL, Rizzoli R, Bonjour JP (1992) Parathyroid hormone-related protein-production by primary cultures of mammary epithelial cells. *J Cell Physiol* **150**: 304–311

Francini G, Maioli E, Petrioli R, Paffetti P, Gonnelli S, Aquino A (1993) Production of parathyroid hormone and parathyroid hormone-related protein by breast cancer cells in culture. *J Cancer Res Clin Oncol* **119**: 421–425

Goltzman D, Hendy GN, Banville D (1989) Parathyroid hormone-like peptide: molecular characterization and biological properties. *Trends Endocrinol Metab* **1**: 39–44

Haq M, Kremer R, Goltzman D, Rabbani SA (1993) A vitamin-D analog (EB 1089) inhibits parathyroid hormone-related peptide production and prevents the development of malignancy-associated hypercalcemia invivo. *J Clin Invest* **91**: 2416–2422

Iwamura M, Abrahamsson PA, Foss KA, Wu G, Cockett ATK, Deftos LJ (1994) Parathyroid hormone-related protein – a potential autocrine growth regulator in human prostate cancer cell-lines. *Urology* **43**: 675–679

Martin TJ, Suva LJ (1989) Parathyroid hormone-related protein in hypercalcemia of malignancy. *Clin Endocrinol* **31**: 631–647

Moniz C, Burton PBJ, Malik AN, Dixit M, Banga JP, Nicolaides K, Quirke P, Knight DE, McGregor AM (1990) Parathyroid hormone-related peptide in normal human fetal development. *J Mol Endocrinol* **5**: 259–266

Powell GJ, Southby J, Danks JA, Stillwell RG, Hayman JA, Henderson MA, Bennett RC, Martin TJ (1991) Localization of parathyroid hormone-related protein in breast cancer metastases – increased incidence in bone compared with other sites. *Cancer Res* **51**: 3059–3061

Schofl C, Cuthbertson KSR, Gallagher JA, Pennington SR, Cobbold PH, Brabant G, Hesch RD, Muhlen AV (1991) Measurement of intracellular $Ca^{2+}$ in single aequorin-injected and suspensions of FURA-2-loaded ROS-17/2.8 cells and normal human osteoblasts – effect of parathyroid hormone. *Biochem J* **274**: 15–20

© Cancer Research Campaign 1997 

Southby J, Kissin MW, Danks JA, Hayman JA, Moseley JM, Henderson MA, Bennett RC, Martin TJ (1990) Immunohistochemical localization of parathyroid hormone-related protein in human breast cancer. *Cancer Res* **50**: 7710–7716

Thiede MA, Rodan GA (1988) Expression of a calcium-mobilising parathyroid hormone-like peptide in lactating mammary tissues. *Science* **242**: 278–280

Vargas SJ, Gillespie MT, Powell GJ, Southby J, Danks JA, Moseley JM, Martin TJ (1992) Localization of parathyroid hormone-related protein messenger RNA expression in breast cancer and metastatic lesions by in situ hybridization. *J Bone Min Res* **7**: 971–979

Walsh CA, Gallagher JA, Luparello C, Pucci-Minafra I, Minafra S (1992) PTHrP production by human breast cancer cells lines in vitro (abstract). *Bone* **13**: 107

Wysolmerski J, Daifotis A, Broadus A, Milstone L, Philbrick W (1993) Overexpression of PTHrP in transgenic mice results in breast hypoplasia. *J Bone Min Res* **8**: S149–S149

 *© Cancer Research Campaign 1997*